# Joint Loads in Marsupial Ankles Reflect Habitual Bipedalism versus Quadrupedalism

**DOI:** 10.1371/journal.pone.0058811

**Published:** 2013-03-12

**Authors:** Kristian J. Carlson, Tea Jashashvili, Kimberley Houghton, Michael C. Westaway, Biren A. Patel

**Affiliations:** 1 Institute for Human Evolution, University of the Witwatersrand, Johannesburg, South Africa; 2 Department of Anthropology, Indiana University, Bloomington, Indiana, United States of America; 3 Department of Geology and Paleontology, Georgian National Museum, Tbilisi, Georgia; 4 Cultures and Histories Program, Queensland Museum, Brisbane, Australia; 5 Department of Cell and Neurobiology, Keck School of Medicine, University of Southern California, Los Angeles, California, United States of America; Raymond M. Alf Museum of Paleontology, United States of America

## Abstract

Joint surfaces of limb bones are loaded in compression by reaction forces generated from body weight and musculotendon complexes bridging them. In general, joints of eutherian mammals have regions of high radiodensity subchondral bone that are better at resisting compressive forces than low radiodensity subchondral bone. Identifying similar form-function relationships between subchondral radiodensity distribution and joint load distribution within the marsupial postcranium, in addition to providing a richer understanding of marsupial functional morphology, can serve as a phylogenetic control in evaluating analogous relationships within eutherian mammals. Where commonalities are established across phylogenetic borders, unifying principles in mammalian physiology, morphology, and behavior can be identified. Here, we assess subchondral radiodensity patterns in distal tibiae of several marsupial taxa characterized by different habitual activities (e.g., locomotion). Computed tomography scanning, maximum intensity projection maps, and pixel counting were used to quantify radiodensity in 41 distal tibiae of bipedal (5 species), arboreal quadrupedal (4 species), and terrestrial quadrupedal (5 species) marsupials. Bipeds (*Macropus* and *Wallabia*) exhibit more expansive areas of high radiodensity in the distal tibia than arboreal (*Dendrolagus*, *Phascolarctos*, and *Trichosurus*) or terrestrial quadrupeds (*Sarcophilus*, *Thylacinus*, *Lasiorhinus*, and *Vombatus*), which may reflect the former carrying body weight only through the hind limbs. Arboreal quadrupeds exhibit smallest areas of high radiodensity, though they differ non-significantly from terrestrial quadrupeds. This could indicate slightly more compliant gaits by arboreal quadrupeds compared to terrestrial quadrupeds. The observed radiodensity patterns in marsupial tibiae, though their statistical differences disappear when controlling for phylogeny, corroborate previously documented patterns in primates and xenarthrans, potentially reflecting inferred limb use during habitual activities such as locomotion. Despite the complex nature of factors contributing to joint loads, broad observance of these patterns across joints and across a variety of taxa suggests that subchondral radiodensity can be used as a unifying form-function principle within *Mammalia*.

## Introduction

Eutherian and metatherian (marsupials) lineages diverged approximately 160 million years ago [1]. The latter often serve as phylogenetic controls for understanding morphology-behavior relationships in the former [2]–[5]. Where consistent form-function relationships appear in both groups, unifying principles in mammalian physiology, morphology and behavior can be established [6]. For example, arboreal quadrupedal marsupials, such as opossums [4], [7] and the tree kangaroo (*Dendrolagus*; [8]) converge on aspects of gait (e.g., compliancy) that are exhibited by arboreal quadrupedal eutherian mammals, such as primates [9]–[10]. Inconsistencies, however, are to be found as well. Some bipedal macropods (e.g., a few kangaroos and wallabies) are uniquely specialized compared to quadrupedal mammals, including the only living arboreal macropod (*Dendrolagus*), in that the former are able to decouple speed and cost of transport [11]–[12]. Moreover, the hind limbs of bipedal macropods appear to experience peak vertical components of the substrate reaction force (SRF) that are twice those experienced by hind limbs of quadrupeds trotting at physiologically equivalent speeds [13].

Eutherian mammals and marsupials [14]–[17] have postcranial morphologies that reflect how they use their skeletons during habitual activities such as locomotion. Direct quantification of loads imposed on articular surfaces during locomotion, however, is problematic because the requisite experimental procedures (e.g., load cells, strain gages) necessitate disruption of joint integrity, which in turn causes abnormal movement. Internal characteristics of the bone that occurs in joints, such as radiodensity of subchondral bone lining some articular surfaces, offer a non-invasive alternative for estimating joint loads [18]–[23], providing otherwise unobtainable information on the form-function relationships expressed in the skeleton of free-ranging mammals. Marsupial joint loads in comparison to those of eutherian mammals are poorly documented, despite the mechanics of some marsupial gaits, particularly hopping, being well-studied [2], [5]–[8], [11], [24]–[29]. Data on marsupial joint loads would provide crucial insight into their intertwined morphology and behavior (e.g., locomotion). Here, we test whether radiodensity patterns in the subchondral bone of marsupial distal tibiae differ in a predictable fashion by evaluating a number of marsupial species characterized by different habitual behavioral activities.

Compressive strength of subchondral bone is determined by mineral content and porosity [30], which can be cumulatively quantified as radiodensity using radiographic-based techniques, such as computed tomography (CT). Higher radiodensity indicates higher apparent density, whether it is through lower porosity, higher mineral content, or a combination of both. When a joint is congruent its articular surfaces are subjected to trivial bending loads; though joint surfaces may experience shear forces, subchondral radiodensity is a reasonable estimator of compressive loads in habitual joint loading regimes in such cases [31]–[32]. Joint congruency is relative and can be dynamic over a range of motion (e.g., close-packed versus not close-packed), with examples such as the humeroulnar joint considered relatively incongruent [32] compared to other joints (e.g., wrist, ankle, and knee joints). Subchondral bone in relatively congruent diarthrodial joints experiences compression from joint reaction forces during behaviors in which the limbs are used, most often during locomotor activities [33]. When limbs are weight-bearing and positioned beneath the body, these joints experience reaction forces that are largely axially-directed and proportional to body weight. Vertical shifts in the center of mass result in accelerations or decelerations of body weight; are partly a function of limb kinematics, substrate use, and speed [34]; and can influence joint loads. Musculotendon complexes bridging a joint also contribute to compressive loads experienced in subchondral bone whenever muscles contract. Studies of cat or rabbit [35] and human knees [36] demonstrate that muscle contractile forces contribute more to overall knee joint loads than body weight. Interestingly, studies of subchondral radiodensity in the distal radius of suspensory and quadrupedal primates and xenarthrans [18]–[20] suggest that muscle contractile forces may contribute less to overall wrist joint loads than body weight [18]–[20]. To date, these relationships providing information about bone functional adaptations are unassessed in the ankle joint. Adding data from the ankle to the burgeoning literature on joint loading, therefore, could help to understand these differences.

Primates and xenarthrans exhibit subchondral radiodensity patterns that corroborate theoretical expectations of limb loading according to their habitual activity patterns (e.g., distributions in forelimbs and hind limbs distinguish bipeds, suspensory, and arboreal or terrestrial quadrupeds). Human distal radii exhibit lower weight-bearing (compressive) loads compared to suspensory and quadrupedal primates, as would be expected since human forelimbs no longer have an active weight-bearing role in locomotion [18]. Suspensory primates, such as orangutans, load their distal radii during locomotion, but since pronograde quadrupedalism comprises such a small percentage of total locomotor repertoires of orangutans compared to percentage of below-branch activity (e.g., suspension) in which the center of mass is below the handhold [37], compressive forces through the orangutan forelimb likely reflect predominantly muscle contractile forces maintaining joint integrity. A similar trend has been documented in xenarthran distal radii when comparing suspensory sloths and quadrupedal anteaters [20]. Quadrupedal primates exhibit the most extensive distribution of high radiodensity in their distal radii, even differing in predictable ways according to hand postures habitually adopted during quadrupedalism [19]. Similarly, the distribution of high radiodensity areas in the primate foot (e.g., calcaneocuboid joint) corroborates theoretical differences in loading arising from overall foot mobility and habitual foot postures adopted during quadrupedalism [22].

The goals of this study are two-fold. First, we aim to document whether a form-function relationship exists in radiodensity patterns in subchondral bone of marsupial distal tibiae. Second, we evaluate whether radiodensity patterns of subchondral bone exhibit a unifying form-function principle within *Mammalia*. In order to achieve these aims, we test two predictions. First, bipedal marsupials should exhibit significantly more expansive high radiodensity area in the distal tibia compared to quadrupedal marsupials because of the unique mechanics of their hopping gait, and because they carry body weight only through the hind limbs rather than all four limbs (i.e., we hypothesize compressive loads in ankles should be proportionately higher in the former group). Second, high radiodensity area in distal tibiae of terrestrial quadrupedal marsupials should exceed high radiodensity area in distal tibiae of arboreal quadrupedal marsupials because the latter may systematically adopt a more compliant gait, and/or systematically use more compliant arboreal substrates (i.e., we hypothesize compressive loads in ankles should be proportionately higher in the former group). If observed relationships between high radiodensity patterns and limb use in marsupials are consistent with previously documented relationships in eutherian mammals (e.g., primates and xenarthrans), it would be reasonable to elevate these relationships to unifying form-function principles throughout *Mammalia*.

## Materials and Methods

The study sample consists of data derived from 41 marsupial tibiae housed in collections curated at the: Australian Museum (AM), Australian National Wildlife Collection (ANWC), Queensland Museum (QM), and Tasmanian Museum (TM) (see Table S1 for specimen information). Either a left or a right tibia was used without preference, often the choice being dictated by availability. We excluded tibiae that originated from known captive individuals in order to avoid sampling idiosyncratic locomotor activities or possibly implicating the use of artificial substrates (e.g., floors of captive enclosures). Data were acquired from specimens only when they lacked obvious visible evidence of trauma or pathology on limb bones, since this could signal potentially altered gait patterns or bone density (e.g., degenerative joint disease), respectively. Institutions allowed short-term loans (e.g., a few hours) for the purposes of transporting specimens to and from scanning facilities, in some cases with a museum representative assisting with the scanning.

We acquired image data from distal tibiae using CT osteoabsorptiometry (Table 1). The data acquisition protocol and rationale have been published elsewhere [18]–[19]. Briefly, tibiae were aligned with their longitudinal axis perpendicular to the scan plane, usually with the anterior surface facing upwards and the tibial plafond positioned in the coronal plane. In some cases, it was necessary to place a tibia with a different surface facing upwards in order to achieve a stable resting position on the CT scanner bed. Such alignment changes have trivial affects since specimens can be repositioned in a virtual 3D environment [18]–[19], but were necessary to avoid movement artifacts that can result from poor stability during CT scanning. To the extent permitted by CT manufacturer and model differences, we attempted to use similar scan parameters in each facility: tube voltage = 120 kV; tube current = 200–300 mA; slice thickness = 0.5–0.625 mm; field of view (FOV) = 180–285; reconstruction increment = 0.4 mm; 512×512 voxel matrix. Image data were reconstructed from raw CT data using standard and bone (e.g., edge-enhanced) filters to produce two sets of DICOM files for each specimen. Image data can be obtained by contacting the corresponding author.

**Table 1:** Sample (n = 41).

Genus	Species	Common name	Tibiae	Source^1^	Gait category
*Macropus*	*giganteus*	Eastern Grey Kangaroo	5	AM, ANWC, QM, TM	Bipedal
*Macropus*	*fuliginosus*	Western Grey Kangaroo	2	ANWC	Bipedal
*Macropus*	*eugenii*	Tammar Wallaby	4	AM, ANWC	Bipedal
*Macropus*	*parma*	Parma Wallaby	1	AM	Bipedal
*Wallabia*	*bicolor*	Swamp Wallaby	2	AM	Bipedal
*Dendrolagus*	*dorianus*	Doria’s Tree-kangaroo	1	ANWC	Arboreal quadrupedal
*Dendrolagus*	*lumholtzi*	Lumholtz’s Tree kangaroo	1	QM	Arboreal quadrupedal
*Phascolarctos*	*cinereus*	Koala	9	AM, ANWC, QM	Arboreal quadrupedal
*Trichosurus*	*vulpecula*	Common Brushtail Possum	6	AM, ANWC, QM	Arboreal quadrupedal
*Lasiorhinus*	*krefftii*	Northern Hairy-nosed Wombat	2	QM	Terrestrial quadrupedal
*Lasiorhinus*	*latifrons*	Southern Hairy-nosed Wombat	1	QM	Terrestrial quadrupedal
*Sarcophilus*	*harrisii*	Tasmanian Devil	1	AM	Terrestrial quadrupedal
*Thylacinus*	*cynocephalus*	Tasmanian Wolf	3	TM	Terrestrial quadrupedal
*Vombatus*	*ursinus*	Common Wombat	3	ANWC, QM, TM	Terrestrial quadrupedal

1 AM: Australian Museum (Sydney); ANWC: Australian National Wildlife Collection (Canberra); QM: Queensland Museum (Brisbane); TM: Tasmanian Museum (Hobart). See Table S1 for specimen numbers.

**Figure 1:**
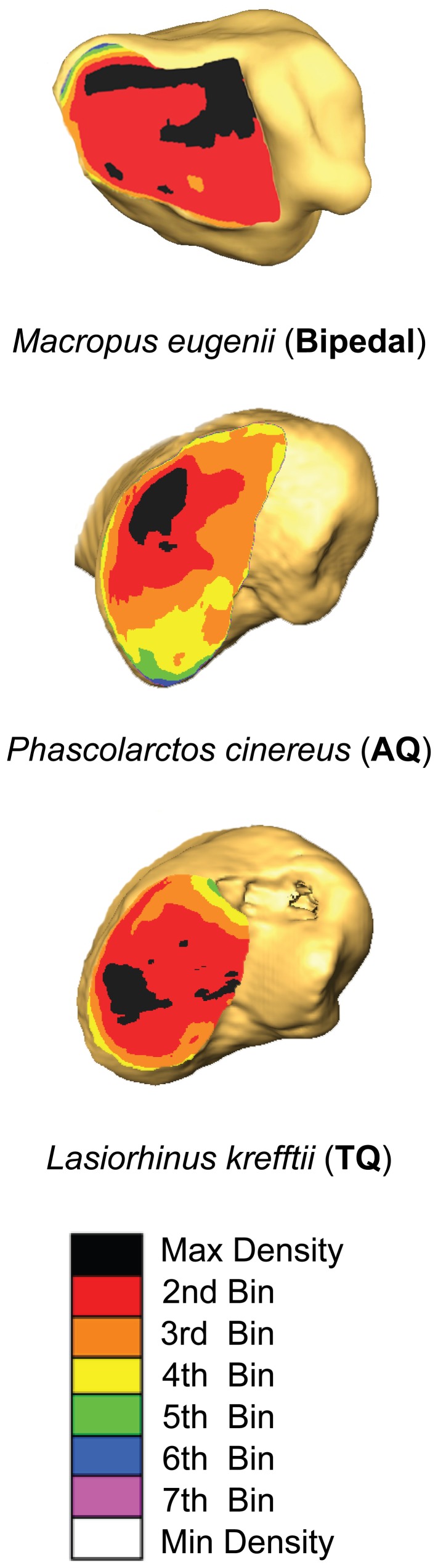
Maximum intensity projection (MIP) maps of marsupials representing gait categories. For each of the distal tibiae, its anterior surface faces upwards and its medial surface faces to the right. AQ = Arboreal quadrupedal, TQ = Terrestrial quadrupedal.

We generated maximum intensity projection (MIPs) maps from the standard reconstructions and fitted them to 3D renderings of distal tibiae generated from the bone reconstructions [18]–[19]. During fitting procedures, we excluded regions of the distal articular surface that descended inferiorly onto the medial malleolus because theoretically this region should not experience substantial axial compressive loading during body weight support by the hind limb (Figure 1). In some cases when this surface is more obliquely oriented rather than vertically oriented, it is reasonable to assume that this surface may experience a relatively small component of axial compressive loading accompanying shear loading. An element of compressive loading also may be experienced by this part of the articular surface due to non-trivial mediolaterally-directed forces during locomotion [38], for example, those possibly arising during movement across uneven terrain. In order to simplify our model of the distal tibia, we focus on the horizontally-positioned portion of the tibiotalar joint, specifically the tibial plafond, and assume that axial loading of the distal tibial articular surface is the primary contributing factor to its compressive loading. Fitting MIPs to 3D renderings was performed in Adobe Photoshop (5.0; Adobe Systems Incorporated). Fitted MIPs represent a 3D voxel matrix that is condensed into a 2D pixel matrix (an 8-bit image) containing the highest radiodensity value extracted from a column of voxels following a line of sight through the depth of the subchondral bone. Pixels in fitted MIPs were binned into eight groups according to gray values, from which false color maps were generated to visualize radiodensity patterns (Figure 1).

Following field observations and locomotor descriptions [39], taxa were parsed into one of three habitual gait categories: bipedal, arboreal quadrupedal (AQ), and terrestrial quadrupedal (TQ). In order to control for potential body size-related differences in pixel counts (i.e., larger specimens potentially have more pixels per bin overall than smaller specimens), we created ratios between the number of pixels in each bin and the total number of pixels in the fitted MIP (i.e., the entire articular surface). Only ratios from the maximum and second highest bins were reported (Table 2), since other bins are less informative to compressive loading patterns [18].

**Table 2:** Descriptive statistics for ANOVA.

		Max/total pixels	Max/total pixels	Second/total pixels	Second/total pixels
Gait category^1^	n	Mean (1 SD)	Range	Mean (1 SD)	Range
Bipedal	14	0.214 (0.151)	0.045–0.567	0.523 (0.156)	0.253–0.799
AQ	17	0.080 (0.103)	0.000–0.352	0.289 (0.158)	0.013–0.580
TQ	10	0.083 (0.077)	0.000–0.174	0.364 (0.203)	0.094–0.667

1 AQ = Arboreal quadrupedal; TQ = Terrestrial quadrupedal. SD = standard deviation.

Ratio distributions for gait groups did not differ significantly from normal distributions according to a series of Kolmogorov-Smirnov Tests (p>0.05). Thus, we applied a series of one-way ANOVAs in order to statistically evaluate observed differences between gait group means (Table 2). Gait group variances did not differ significantly according to Levene tests for homogeneity of variances (p>0.05). Thus, in order to evaluate the statistical significance of pairwise comparisons, we applied a series of Tukey Honestly Significant Difference post-hoc tests. We used SPSS (v. 16.0.1) for these statistical analyses.

**Figure 2:**
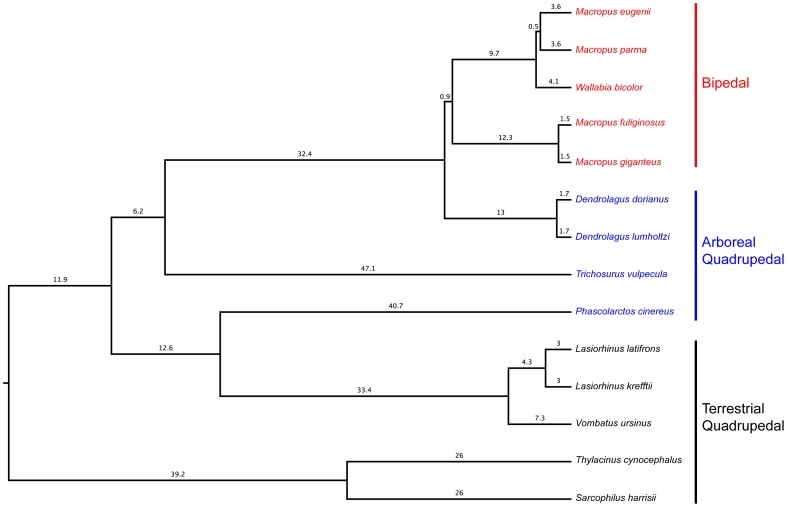
Phylogenetic tree and divergence times of marsupial species included in the study. Branch lengths indicate millions of years based on molecular analyses [40]–[41]. Colors distinguish between groups characterized by different habitual locomotor activities (red: bipedal; blue: arboreal quadruped; black: terrestrial quadruped).

In our sample, some marsupial taxa representing gait groups are also closely related (e.g., bipeds are all macropods). In order to tease apart the extent to which observed gait group differences may have reflected phylogeny and function, we performed a second set of ANOVAs in which we accounted for phylogenetic effects (pANOVAs). We constructed a phylogenetic tree with known divergence dates (Figure 2) for taxa in the sample based on recent molecular studies of marsupials [40]–[41]. The tree file was created with Mesquite software (Table S2). For pANOVAs and post-hoc analyses, we used R software and the *phytools* package [42]. In all statistical testing, significance was established at p<0.05.

## Results

### Prediction 1: Bipedal Marsupials>Quadrupedal Marsupials

**Figure 3:**
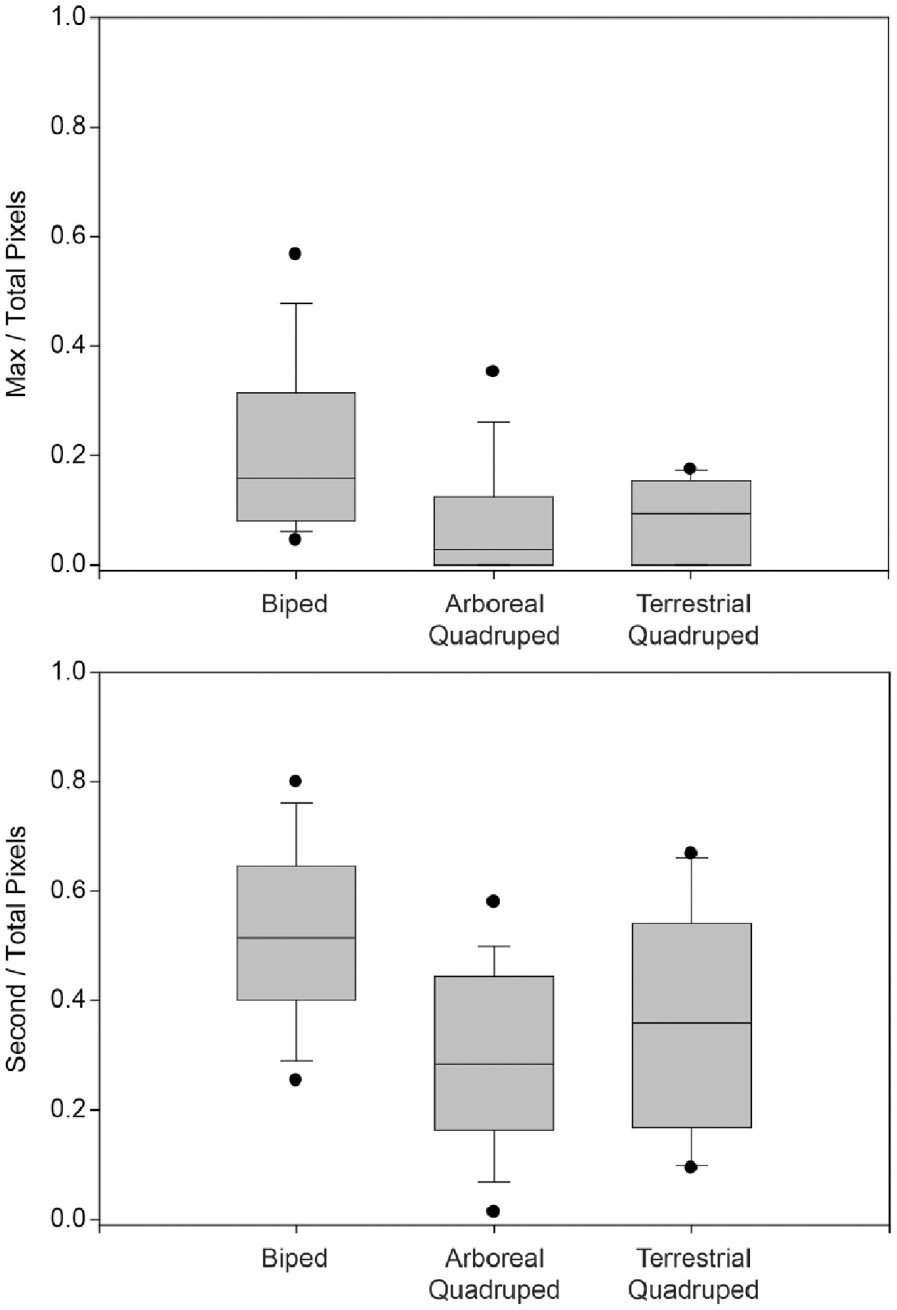
Box plots of pixel ratios in articular surfaces. Top row indicates ratios of pixels in the maximum bin to total pixels, while the bottom row indicates ratios of pixels in the second highest bin to total pixels. These values are derived from distal tibiae, excluding the region of the articular surface that descends onto the lateral aspect of the medial malleolus. Horizontal lines within each box indicate medians, while the entire box envelops the interquartile range of the distribution (i.e., 50% of values), and whiskers encompass the remaining range, excluding outliers. Filled circles beyond whiskers indicate outliers.

Ratios of maximum radiodensity area to total articular surface area differ between groups. As predicted, bipeds exhibit the largest ratios (Figure 3, Tables 2 and 3), regardless of whether they are small-bodied (wallaby mean = 0.184, n = 7: Tables 1 and 3) or large-bodied (kangaroo mean = 0.243, n = 7: Tables 1 and 3). Biped ratios are significantly higher than ratios of quadrupeds, whether they are arboreal or terrestrial (Table 4). Interestingly, the only arboreal macropod (*Dendrolagus*) has a comparatively low mean ratio of 0.017 (n = 2). When accounting for phylogeny, the comparison of species means eliminates statistical significance (Table 4).

**Table 3:** Descriptive statistics for pANOVA.

			Max/total pixels	Second/total pixels
Genus^1^	Species	n	Mean (1 SD)	Mean (1 SD)
*Macropus*	*giganteus*	5	0.298 (0.203)	0.470 (0.104)
*Macropus*	*fuliginosus*	2	0.107 (0.043)	0.532 (0.271)
*Macropus*	*eugenii*	4	0.149 (0.077)	0.562 (0.229)
*Macropus*	*parma*	1	0.076 (−)	0.668 (−)
*Wallabia*	*bicolor*	2	0.307 (0.003)	0.495 (0.018)
**Bipedal species**		**5**	**0.187 (0.108)**	**0.545 (0.077)**
*Dendrolagus*	*dorianus*	1	0.029 (−)	0.144 (−)
*Dendrolagus*	*lumholtzi*	1	0.005 (−)	0.457 (−)
*Phascolarctos*	*cinereus*	9	0.110 (0.127)	0.272 (0.145)
*Trichosurus*	*vulpecula*	6	0.058 (0.065)	0.309 (0.188)
**AQ species**		**4**	**0.051 (0.045)**	**0.296 (0.129)**
*Lasiorhinus*	*krefftii*	2	0.138 (0.019)	0.635 (0.046)
*Lasiorhinus*	*latifrons*	1	0.000 (−)	0.094 (−)
*Sarcophilus*	*harrisii*	1	0.000 (−)	0.153 (−)
*Thylacinus*	*cynocephalus*	3	0.133 (0.060)	0.355 (0.155)
*Vombatus*	*ursinus*	3	0.051 (0.086)	0.353 (0.167)
**TQ species**		**5**	**0.064 (0.068)**	**0.318 (0.212)**

1 AQ = Arboreal quadrupedal; TQ = Terrestrial quadrupedal. SD = standard deviation. See Table 1 for common names.

**Table 4:** ANOVA and pANOVA results.

					ANOVA				pANOVA
Ratio	Comparison^1^	df	F	p	Post hoc analyses	df	F	p	Post hoc analyses
Max bin/total	B v. AQ v. TQ	2, 38	5.920	0.006**	B v. AQ: p = 0.008**	2, 11	4.152	0.045*	B v. AQ = 0.342
					B v. TQ: p = 0.027*			0.422^2^	B v. TQ: p = 0.698
					AQ v. TQ: p = 0.998				AQ v. TQ: p = 0.903
Second bin/total	B v. AQ v. TQ	2, 38	7.492	0.002**	B v. AQ: p = 0.001**	2, 11	3.948	0.051	B v. AQ = 0.441
					B v. TQ: p = 0.072			0.410^2^	B v. TQ: p = 0.760
					AQ v. TQ: p = 0.511				AQ v. TQ: p = 0.907

1 B = Bipedal; AQ = Arboreal quadrupedal; TQ = Terrestrial quadrupedal.

2 Phylogenetic p-value. df = Degrees of Freedom;

* denotes statistical significance at 0.05 level;

** denotes statistical significance at 0.01 level.

Ratios of the second highest radiodensity area to total articular surface area differ between groups in a way that is consistent with differences in maximum-to-total ratios. Bipeds again exhibit the largest ratios (Figure 3, Tables 2 and 3), regardless of whether they are small-bodied (wallaby mean = 0.558, n = 7: Tables 1 and 3) or large-bodied (kangaroo mean = 0.488, n = 7: Tables 1 and 3). The biped ratio is significantly higher than the quadruped ratio, but only the difference between bipeds and arboreal quadrupeds is significant while the difference between bipeds and terrestrial quadrupeds is borderline non-significant (Table 4). The only arboreal macropod (*Dendrolagus*) again has a comparatively low mean ratio of 0.300 (n = 2) compared to other macropods. When accounting for phylogeny, the comparison of species means eliminates statistical significance (Table 4).

### Prediction 2: Terrestrial Quadrupedal Marsupials>Arboreal Quadrupedal Marsupials

As predicted, arboreal quadrupeds exhibit the smallest maximum radiodensity area relative to total articular surface area of any group (Figure 3, Tables 2 and 3). However, differences between terrestrial quadruped and arboreal quadruped ratios are small and non-significant (Table 4). When accounting for phylogeny, the comparison of species means remains non-significant (Table 4).

Arboreal quadrupeds also exhibit the smallest ratios of second highest radiodensity area to total articular surface area of any group in a way that is consistent with maximum-to-total ratios (Figure 3, Tables 2 and 3). As was observed in comparisons of maximum radiodensity area ratios, however, differences between terrestrial quadruped and arboreal quadruped ratios are small and non-significant (Table 4). When accounting for phylogeny, the comparison of species means remains non-significant (Table 4).

## Discussion

As predicted, bipedal marsupials such as kangaroos and wallabies exhibit distal tibiae with more expansive maximum radiodensity areas than the distal tibiae of quadrupedal marsupials investigated in this study. It appears that compressive loads borne through hind limbs are more substantial in bipeds than quadrupeds, possibly due to more body weight support per hind limb generating higher vertical components of SRFs experienced in the former [13]. While we did not exhaustively sample bipedal and quadrupedal marsupials, the extent of the observed differences in representative taxa (Tables 1 and 3) strongly suggests that this may be a common trend amongst marsupials. The statistical significance of this trend disappears when accounting for phylogeny (Table 4). However, it is noteworthy in support of a functional basis to the observed trend that two species of *Dendrolagus*, the tree kangaroo, and the only living arboreal macropod, exhibit substantially lower mean ratios compared to mean ratios of closely related bipedal macropods (Figure 2 and Table 3). This suggests a functional signal in macropod distal tibiae is not completely obscured by phylogeny. Moreover, the phylogenetically-controlled ANOVAs, which utilize species means and thus do not account for intraspecific biological variation, are hampered by small sample sizes in several cases. In our study, *Macropus parma*, *Sarcophilus harrisii*, and *Lasiorhinus latifrons*, each represented by only one individual per species, have notably low values compared to related taxa that are represented by multiple individuals (Table 4). Investigation of radiodensity patterns within primate distal tibiae would be a worthwhile investigation as an independent test of the bipedal versus quadrupedal signal that is observed in the present study. While there are clear differences between the mechanics of macropod and human bipedalism, based on the present results we would predict that patterns in primate distal tibiae should mirror those in marsupials, specifically, that bipedal humans should be unique (and more expansive) in relative maximum radiodensity area compared to quadrupedal primates.

The distinctiveness of distal tibiae of bipedal marsupials corroborates findings of others who have noted distinctiveness of the calcaneus of bipedal marsupials [43]. Functional anatomy of the marsupial calcaneus, including its dimensions, would seem strongly integrated with functional anatomy of the ankle joint. Serving as the insertion point for the Achilles tendon, and thus operating as the moment arm for ankle plantar flexors such as the triceps surae, the calcaneus plays a key role in foot function. Among several hind limb joints of tammar wallabies (*Macropus eugenii*), the ankle was observed to be the greatest contributor to whole limb work and power over a range of accelerations and decelerations [44]. The present study provides supporting evidence for the ankle joint playing a central role in the distinctiveness of macropod hopping gaits versus gaits of other marsupials. In addition to the distal tibia, it would be worthwhile to assess the articulating component of the ankle joint (i.e., the talar trochlea) for concordant or discrepant trends, as has been done in some primates [23].

It was unexpected that mean radiodensity ratios of arboreal and terrestrial quadruped marsupials did not differ significantly. Arboreal quadrupedal primates exhibit gait compliancy compared to terrestrial quadrupedal primates, and thus limbs of the former experience lower peak vertical components of SRFs [9]–[10], [45]. A convergent pattern in aspects of gait compliancy has been demonstrated in at least one arboreal quadrupedal marsupial [4]. Arboreal substrates usually are more compliant and more susceptible to movement (i.e., instability) compared to terrestrial substrates, which could favor reduced peak compressive loading experienced through limb joints because of higher duty factors when moving on branches [46]. One possible explanation for this unexpected result amongst marsupials could be that gait compliancy is more effective at reducing compressive loads in forelimb joints rather than hind limb joints, as appears to be the case in some primates. In capuchin monkeys, for example, peak vertical components of SRFs experienced by their forelimbs during walks, runs, and gallops were significantly lower on simulated arboreal supports than on a wooden runway, while hind limb peak vertical forces did not differ significantly between substrates during as many of these gaits [47]. It would be worthwhile, in this regard, to compare marsupial distal radii from the same locomotor groups and document whether bipeds exhibit the smallest areas of maximum radiodensity and terrestrial quadrupeds the largest.

A second potential explanation for the unexpected non-significant difference between arboreal and terrestrial marsupial quadrupeds could be related to the application of such rigid gait and substrate use categories in the first place. There are a wealth of primate observational data on locomotor activities and substrate use [48]. Similar data from free-ranging marsupials are not as widely documented. For example, some primates traditionally recognized as quadrupeds also have been observed habitually using suspensory behaviors [49]. Primates typically categorized as digitigrade or palmigrade have been shown to dynamically switch between both hand postures, depending on speed, gait, and substrate used during quadrupedal gaits [50]–[54]. Because of the relative paucity of quantitative observational data on marsupial locomotor behavior and substrate use, the possibility exists that there could be an underappreciated amount of crossover in gait and substrate use amongst the marsupial quadrupeds included in this study. If arboreal and terrestrial quadrupedal marsupials differ less in substrate preference than presently assumed, it could explain the greater than expected similarity exhibited in their relative maximum radiodensity areas. It is worth noting that compliant gait has been proposed as a solution for additional challenges besides those arising during arboreal locomotion [55]. With additional data on substrate use, reassessing quadrupedal marsupials (or primates) according to terrain unevenness and compliant gait, irrespective of whether they are presently characterized as arboreal or terrestrial, could provide interesting insights for the observed variation in subchondral radiodensity areas and estimated compressive loads of marsupial (or primate) distal tibiae.

Expanding samples of fossorial (i.e., *Vombatus* and *Lasiorhinus*) and non-fossorial terrestrial quadrupeds (i.e., *Sarcophilus* and *Thylacinus*) would facilitate another potentially interesting comparison within marsupials. Wombats are described as emphasizing forelimbs during digging, while their hind limbs are primarily relegated to clearing accumulated sediment [43]. Thus, wombat hind limbs may be less functionally specialized away from the limbs of other terrestrial quadrupeds compared to wombat forelimbs. External calcaneal morphology of fossorial and non-fossorial quadrupeds supports this possibility since their calcanei could not be differentiated in a morphometric analysis of calcaneal shape and dimension [43]. As noted previously, investigating the distal radius of bipedal and different forms of quadrupedal marsupials could prove especially illuminating. We would predict that fossorial taxa may show evidence of elevated compressive loading of the wrist compared to all other marsupial taxa, if digging indeed fosters more morphological specialization in the forelimb than the hind limb.

We are aware of only one published study that has focused on quantifying similar radiodensity patterns in joints of the foot. Nowak et al. [22] observed the predicted correspondence between presumed joint loading patterns and locations of maximum radiodensity areas in primates that experience a “mid-tarsal break” versus humans that typically do not, but that may in small frequencies [56]. Observation of the predicted distinctiveness of bipedal marsupials in the extent of maximum radiodensity area in their distal tibiae also accords well with predicted patterns of radiodensity distribution observed in the forelimb joints of primates and xenarthrans. Quadrupedal primates exhibit more expansive areas of high radiodensity in their distal radius compared to the distal radii of bipedal and suspensory primates [18], while quadrupedal xenarthrans exhibit more expansive areas of high radiodensity in their distal radius compared to the distal radii of suspensory sloths [20]. In each case, it is reasonable to conclude that the quadrupedal group experiences compressive loading during forelimb use resulting from the *additive* effects of body weight support and musculotendon contractile forces from complexes that bridge joints, while the other groups may experience the former contributing factor to a lesser degree. Teasing apart the relative contribution of the two factors is complex, however, as demonstrated by a recent study of joint forces in the human knee showing that a muscle can contribute to overall joint force via its contribution to the substrate reaction force even when the muscle does not cross the joint in question [57].

Despite the limitations of this study (e.g., small samples of some species, relative lack of quantitative data on marsupial behavior and substrate use, and the complexity of musculotendon loading of joints), the observed trend exhibited by bipedal and quadrupedal marsupials adds to the accumulating evidence suggesting that the relationship between relative area of maximum radiodensity and joint compressive loads in limbs may be a unifying form-function principle amongst eutherian and metatherian mammals. By demonstrating how behavior influences morphology in a variety of mammals, these unifying principles support the existence of form-function signals in skeletal tissues that often elude comparative functional anatomists.

## Supporting Information

Table S1 Specimen information and raw data used in analyses.
(XLS)

Table S2 Nexus tree file used in the pANOVA. The phylogenetic tree with known divergence dates for taxa in the sample is based on recent molecular studies of marsupials [40]–[41]. The tree file was created with Mesquite software.
(NEX)
